# An approach using ensemble empirical mode decomposition to remove noise from prototypical observations on dam safety

**DOI:** 10.1186/s40064-016-2304-4

**Published:** 2016-05-17

**Authors:** Huaizhi Su, Hao Li, Zhexin Chen, Zhiping Wen

**Affiliations:** State Key Laboratory of Hydrology-Water Resources and Hydraulic Engineering, Hohai University, Nanjing, 210098 China; College of Water Conservancy and Hydropower Engineering, Hohai University, Nanjing, 210098 China; National Engineering Research Center of Water Resources Efficient Utilization and Engineering Safety, Nanjing, 210098 China; Department of Computer Engineering, Nanjing Institute of Technology, Nanjing, 211167 China

**Keywords:** Dam safety, Prototypical observations, Noise removal, Ensemble empirical mode decomposition

## Abstract

It is very important for dam safety control to identify reasonably dam behavior according to the prototypical observations on deformation, seepage, stress, etc. However, there are many cases in which the noise corrupts the prototypical observations, and it must be removed from the data. Considering the nonlinear and non-stationary characteristics of data series with signal intermittency, an ensemble empirical mode decomposition (EEMD)-based method is presented to remove noise from prototypical observations on dam safety. Its basic principle and implementation process are discussed. The key parameters and rules, which can adapt the noise removal requirements of prototypical observations on dam safety, are given. The displacement of one actual dam is taken as an example. The noise removal capability of EEMD-based method is assessed. It is indicated that the dam displacement feature can be reflected more clearly by removing noise from prototypical observations on dam displacement. The statistical model, which is built according to noise-removed data series, can provide the more precise forecast for structural behavior.

## Background

Due to its public and economic impacts and consequences, safety of a dam is of high priority. Based on the prototypical observations of dam body, dam foundation, high slope, surrounding environment, and impact on reservoir dam due to landslides (Pudasaini 2014; Kafle et al. 2016) and seepage (Pudasaini 2016), some mathematical, mechanical and artificial intelligence theories and methods are usually used to analyze and evaluate the dam behavior. It is regarded as an effective approach ensuring service safety of dam engineering (Su et al. 2011). Noise, which can be caused by environmental, man-made and other uncertain factors, is an inevitable part of prototypical observations. The true characteristics of dam behavior sometimes even cannot be reflected from noisy observations. Moreover, the noise has certain effect on further data analysis precision. So some smoothing or filtering methods for noisy data are usually adopted to implement the noise removal of prototypical observations.

At present, wavelet methods are regarded as a powerful alternative tool for removing noise (Shark and Yu 2000; Athanasia and Theofanis 2011; Mohideen 2012). The wavelet coefficients of signal and noise have different characteristics at each wavelet scale. The appropriate wavelet basis function and decomposition layer number are determined according to analyzed signals. The reconstruction of decomposed signals is implemented to fulfill the noise removal. These methods have been widely applied to data pretreatment. However, it is well known that the basis function needs to be fixed in advance for implementing wavelet analysis. It is difficult to approximate accurately the local signal characteristics at different scales with the wavelet function, which is derived from basis function.

Huang et al. (1998) proposed the empirical mode decomposition (EMD) to implement the time–frequency data analysis for nonlinear and non-stationary time series. EMD-based noise removal method has been used recently in many fields such as biology, ocean, medicine, acoustics, fault diagnosis (Huang et al. 1999; Liu et al. 2006; Lee et al. 2011; Park et al. 2011; Ahrabian et al. 2013; Moghtaderi et al. 2013). It does not need to select the basis function in advance and has better adaptive feature. However, when the signal is a superposition of intermittent component and continuous basic component, the unexpected mode mixing will be caused during the mode decomposition. The frequent appearance of mode mixing can make different intrinsic mode function (IMF) components not be effectively separated with EMD. A single IMF component consists of signals of widely disparate scales, or a signal of a similar scale resides in different IMF components. Mode mixing is often a consequence of signal intermittency. The signal intermittency can cause no enough signal extreme points or uneven distribution interval of signal extreme points. Upper and lower envelope generated based on above points is a superposition of intermittent signal envelope and basic signal envelope, which will not only cause serious aliasing in the time–frequency distribution, but also make the physical meaning of individual IMF component unclear.

To overcome the scale separation problem, Wu and Huang (2009) proposed the ensemble empirical mode decomposition (EEMD), which inherits the advantages of EMD. According to the statistical characteristics of Gaussian white noise, namely uniform frequency distribution, a white noise is added to original signal. This method solves the mode mixing problem caused by signal intermittency. The ensemble empirical mode decomposition is introduced to reduce the noise level of prototypical observations on dam safety. This paper is organized as follows. First, the general principle and step of EEMD are reviewed briefly in “Ensemble empirical mode decomposition of nonlinear and non-stationary signal” section. Later, the EEMD-based noise removal process of prototypical observations on dam safety is presented and the algorithm is described in the following section “Noise removal of prototypical observations on dam safety”. In “Actual case analysis” section, the proposed method is applied to noise removal of prototypical observations on one actual dam and statistical model construction. By comparison of fitting and forecasting precision of statistical models before and after noise removal, the validity of proposed method is discussed. Finally, this work briefly concludes in “Conclusions” section.

## Ensemble empirical mode decomposition of nonlinear and non-stationary signal

As an adaptive time–frequency data analysis method, EMD takes a nonlinear and non-stationary signal as integration of some intrinsic mode function (IMF) components. The signal is decomposed layer by layer according to the characteristic scale of signal extrema. A series of IMF components from high frequency to low frequency can be produced, and a residual can be obtained. The handled IMF components are chosen to implement signal reconstruction and fulfill noise removal.

Given a signal *x*(*t*), all local extrema of *x*(*t*) are identified firstly. Cubic spline curves are adopted to fit local minima or local maxima, respectively. Upper and lower envelopes of *x*(*t*) are generated. Secondly, the mean of upper and lower envelopes, *m*_1_(*t*), is calculated. The mean *m*_1_(*t*) is subtracted from *x*(*t*) and the differential signal, *h*_1_(*t*) = *x*(*t*) − *m*_1_(*t*), is obtained where *h*_1_(*t*) is a signal without low frequency. If *h*_1_(*t*) satisfies the IMF condition, then *h*_1_(*t*) is regarded as the first IMF component of the signal *x*(*t*). If not, the second sifting operation needs to be implemented, namely the above procedure for *h*_1_(*t*) needs to be repeated, to obtain *h*_11_(*t*) = *h*_1_(*t*) − *m*_11_(*t*). The sifting process is repeated *j* times, until *h*_1*j*_(*t*) = *h*_1(*j*−1)_(*t*) − *m*_1*j*_(*t*) satisfies the IMF condition. *h*_1*j*_(*t*) is regarded as the first IMF component of the signal *x*(*t*), namely *c*_1_(*t*) = *h*_1*j*_(*t*). Let *r*_1_(*t*) = *x*(*t*) − *c*_1_(*t*). The component *c*_1_(*t*) is extracted from *x*(*t*) and a residual signal *r*_1_(*t*), in which the high frequency component is filtered, is obtained. For *r*_1_(*t*), the above sifting operation is implemented again. Similarly, the second IMF component *c*_2_(*t*) of the signal *x*(*t*) and the residual signal *r*_2_(*t*) are extracted. Such sifting procedure is repeated until the stopping criterion of signal decomposition is satisfied. Once this is achieved, the signal *x*(*t*) can be decomposed adaptively into *n* IMF components from high frequency to low frequency, namely *c*_1_(*t*), *c*_2_(*t*),…, *c*_*n*_(*t*), and a residual *r*_*n*_(*t*),

1 $$x\left( t \right) = \sum\limits_{i = 1}^{n} {c_{i} \left( t \right)} + r_{n} \left( t \right).$$

According to the characteristic scale of signal extrema, the components of the signal *x*(*t*) are decomposed successively from high frequency to low frequency. The residual *r*_*n*_(*t*) is the signal trend component which represents the average trend of the signal *x*(*t*). Thus it can be seen that EMD algorithm has good filtering properties. The decomposition process can be regarded as a filtering process that the characteristic scale of signal extrema is taken as the measure criterion. Furthermore, this algorithm decomposes a signal based on own signal information and the basis function needs to be fixed during signal decomposition. To alleviate the mode mixing problem of EMD, a new noised-assisted data analysis method, namely the ensemble EMD (EEMD), is proposed. The principle of the EEMD is as follows. It defines the true IMF components as the mean of an ensemble of trials, each consisting of the original signal plus a white noise of finite amplitude. The added white noise would populate the whole time–frequency space uniformly with the constituting components of different scales. When the signal is added to this uniformly distributed white background, the signal components with different scales are automatically projected onto proper reference scales established according to the white noise. So the intermittent component of the signal has continuous feature. By adding finite noise, the EEMD eliminates largely the mode mixing problem (Taraphder and Chakraverty 2015).

Given a signal *x*(*t*), the effective algorithm of EEMD can be summarized as follows. Firstly, set the total number (*N*) of added white noise and its amplitude *ε*. Secondly, add the random Gaussian white noise sequence *ω*_*k*_(*t*) to the original signal *x*(*t*). Obtain the noise-added signal *x*_*k*_(*t*), namely

2 $$x_{k} \left( t \right) = x\left( t \right) + \varepsilon \omega_{k} \left( t \right),\quad k = 1,2, \ldots ,N.$$

Thirdly, implement EMD operation for the noise-added signal *x*_*k*_(*t*). Then, obtain *n* IMF components, *c*_*ik*_(*t*), *i* = 1,2,…, *n*, where *c*_*ik*_(*t*) represents the *i*th IMF component obtained with EMD of the signal added *k*th white noise sequence. Lastly, calculate the ensemble mean of each IMF component. The result in the following can be obtained.

3 $$c_{i} \left( t \right) = \frac{1}{N}\sum\limits_{k = 1}^{N} {c_{ik} \left( t \right)} ,\quad i = 1,2, \ldots ,n.$$

**Fig. 1:**
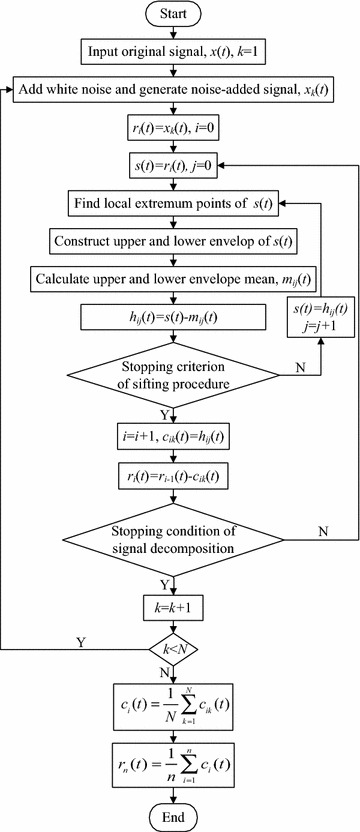
EEMD flowchart

Figure 1 shows the flowchart of EEMD algorithm.

## Noise removal of prototypical observations on dam safety

The prototypical observation series on dam safety has nonlinear and non-stationary characteristics. Most of its information focuses on the low frequency part, and the noise is mainly distributed in the high frequency part. It often contains the intermittent signal. EEMD is introduced to decompose the prototypical observations on dam safety into a series of IMF components from high frequency to low frequency. First few noise-added IMF components are chosen to implement the noise removal with the threshold method, then we reconstruct the noise-removed observation of dam safety

4 $$x^{{\prime }} \left( t \right) = \sum\limits_{i = 1}^{k} {c_{i}^{{\prime }} (t)} + \sum\limits_{i = k + 1}^{n} {c_{i} \left( t \right)} + r_{n} \left( t \right),$$

where *k* denotes the number of IMF components which are chosen to implement the noise removal, *c*_*i*_(*t*) is the IMF component with noise, *c*_*i*_′(*t*) is the noise-removed IMF component, *r*_*n*_(*t*) is a residual.

### Total number of added white noise and its amplitude

For EEMD algorithm, the added white noise has influence on the results, which follows the statistical principle as (Wu and Huang 2009)

5 $$\varepsilon_{n} = {\varepsilon}/{\sqrt{N}},$$

where *ε*_*n*_ is the standard deviation representing the difference between the input signal and the final reconstructed result of IMF components, *ε* denotes the amplitude of added noise, and *N* is the total number of added noise. If the amplitude of added noise is too small, the added noise cannot affect the expected selection of extreme points. Furthermore, if the amplitude of added noise is proper and the number of added noise is enough, the increasement of amplitude and number of added noise has no more effect on the decomposition results. It is suggested that the amplitude of added white noise is taken as 0.2 times of standard deviation of the signal (Wu and Huang 2009). For high frequency component-oriented signal, small amplitude of added noise should be chosen. In general, when the number of added noise is up to 100 or 200, the satisfactory result can obtained.

### Stopping criterion of sifting process

In fact, the EMD is a process sifting IMF components. The stopping criterion of sifting process is used to control the sifting times of generating one IMF component, namely the fulfillment of two conditions in the IMF definition. The too strict stopping criterion will cause the over-sift of IMF components and the elimination of amplitude changes. The easy stopping criterion will lead to the under-sift of IMF components, the riding waves cannot be eliminated and the condition of local zero mean cannot be satisfied. The conventional stopping criteria of sifting process have the standard deviation criterion and overall local combination rule (Huang et al. 1998, 1999). However, based on these stopping criteria, the decomposition process is very sensitive to local disturbance of the signal. The decomposition results of target signals with different local disturbances are very different and irregular. So these conventional stopping criteria of sifting process are not applicable to the EEMD algorithm that the white noises need to be added repeatedly. To overcome this problem, Wu and Huang (2004) proposed the approach fixing the sifting times and they reveal that the upper and lower envelopes of IMF component are almost symmetrical about the zero axis when the sifting times is up to 10.

### Stopping condition of decomposition process

For the EMD algorithm, the decomposition process can be terminated when any following condition is satisfied, namely, the *n*th IMF component *c*_*n*_(*t*) or the residual *r*_*n*_(*t*) is less than the preset value, or the residual *r*_*n*_(*t*) can be regarded as a monotonic function. It is known that for the white noise populating the whole time or frequency space uniformly with the constituting components of different scales, the role of EMD decomposition is equivalent to a binary filter group. The white noise can be decomposed into a series of IMF components with different average periods, and the average period of any IMF is double average period of previous IMF (Flandrin et al. 2004; Wu and Huang 2004). The average period represents the total number of data, namely signal length, divided by the peak point number, or local maximum point number. Therefore, for the EEMD algorithm that the added white noise populates the whole time–frequency space uniformly, the total number *n* of IMF component decomposed completely approximates log_2_*M* − 1, where *M* represents the signal length. In practice, according to the actual requirement, other appropriate conditions can be adopted to terminate the decomposition process. For example, when the extreme point number is less than a certain number, or when the number of IMF component decomposed is up to a certain number, the decomposition process is over.

### Endpoint effect

**Fig. 2:**
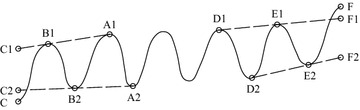
Endpoint effect control scheme

In the sifting process of the EMD algorithm, the extreme points of the signal can be selected to fit the upper and lower envelopes with one cubic spline curve. However, two endpoints of the signal may be not the extreme points. Divergence phenomenon of upper and lower envelopes often appears near two endpoints of the signal, which is called the endpoint effect. Furthermore, this divergence will gradually pollute the whole signal with subsequent sifting process and make the decomposition results distorted seriously. There are two conventional approaches solving the endpoint effect problem of EMD. The first one is that the data near two endpoints are discarded constantly to make the distortion of upper and lower envelope be minimized. The second one is that enough extreme points are obtained by signal extension or forecast. In the sifting process, the maximum and the minimum at the endpoints need to be obtained to make the whole signal be included completely between the fitted envelopes. So a simple and effective method controlling the endpoint effect of EMD is adopted in this paper (Wu and Huang 2009). The connection line between two maximum points near the endpoint is extended to the endpoint. This value is compared with the actual value of the endpoint. The larger one is regarded as the maximum at the endpoint which is used to fit the upper envelope. The connection line between two minimum points near the endpoint is extended to the endpoint. This value is compared with the actual value of the endpoint. The smaller one is regarded as the minimum at the endpoint which is used to fit the lower envelope. Above process is illustrated in Fig. 2. In Fig. 2, A1 and B1 are two maximum points near the left endpoint C of the signal, A2 and B2 are two minimum points near the left endpoint C of the signal. C1 is determined by extending the line A1–B1 to the endpoint. If C1 > C,then C1 is taken as the maximum at the left endpoint. C2 is determined by extending the line A2–B2 to the endpoint. If C < C2,then C is taken as the minimum at the left endpoint. Likewise, F and F2 are determined as the maximum and the minimum at the right endpoint, respectively.

### EEMD-based noise removal process of prototypical observations on dam safety

**Fig. 3:**
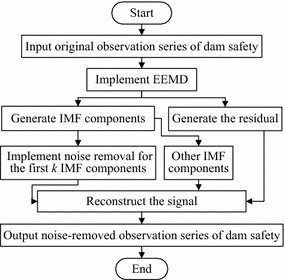
EEMD-based noise removal flowchart

Figure 3 shows an implement process for EEMD-based noise removal of prototypical observations on dam safety. Its key steps are as follows.

#### Implement EEMD

The amplitude of added white noise is taken as 0.2 times of standard deviation of prototypical observation series. The number of added noise is set as 200. The sifting number is set as 10. When the number *n* of IMF component decomposed is up to log_2_*M* − 4, the decomposition process is terminated, where *M* is the length of observation series. EEMD of prototypical observation series on dam safety is fulfilled and *n* IMF components are obtained.

#### Select the IMF components to remove noise

It has been known that, for each IMF component of white noise signal, the product of its energy density and average period is a constant (Wu and Huang 2004). Namely,

6 $$E_{i} \bar{T}_{i} = const,$$

where

7 $$E_{i} = \frac{1}{M}\sum\limits_{t = 1}^{M} {\left[ {c_{i} \left( t \right)} \right]^{2} } ,$$

*E*_*i*_ represents the energy density of the *i*th IMF component *c*_*i*_ of white noise, *M* is the signal length,

8 $$\bar{T}_{i} = {M \mathord{\left/ {\vphantom {M {M_{\hbox{max} } }}} \right. \kern-0pt} {M_{\hbox{max} } }},$$

$\bar{T}_{i}$ represents the average period of *c*_*i*_, *M*_max_ is the number of maximum point of *c*_*i*_.

A statistical magnitude *R*_*k*_ is defined as follows.

9 $$R_{k} = \left| {{{\left( {E_{k + 1} \bar{T}_{k + 1} - E_{k} \bar{T}_{k} } \right)} \mathord{\left/ {\vphantom {{\left( {E_{k + 1} \bar{T}_{k + 1} - E_{k} \bar{T}_{k} } \right)} {\left( {\frac{1}{k}\sum\limits_{i = 1}^{k} {E_{i} \bar{T}_{i} } } \right)}}} \right. \kern-0pt} {\left( {\frac{1}{k}\sum\limits_{i = 1}^{k} {E_{i} \bar{T}_{i} } } \right)}}} \right|,$$

where *E*_*k*_ and $\bar{T}_{k}$ represent, respectively, the energy density and the average period of the *k*th IMF component *c*_*k*_, which is obtained by implementing the EEMD of prototypical observation series on dam safety.

When *R*_*k*_ ≥ *C*, *C* is usually between 2 and 3, most of the noise is contained in the first *k* IMF components. The noise removal for the *k* IMF components need to be implemented.

#### Implement the noise removal with the threshold method

In general, the IMF component with noise contains a small amount of high frequency part of real signal. If the IMF components of certain scales are filtered completely, some useful information may be cleaned, which will affect the accuracy of subsequent analysis. The threshold method is introduced to implement the noise removal for the IMF component *c*_*i*_(*t*). *c*_*i*_′(*t*) represents the noise-removed IMF component.

10 $$c_{i}^{{\prime }} (t) = \left\{ {\begin{array}{*{20}l} {\text{sgn} \left( {c_{i} \left( t \right)} \right)\left( {\left| {c_{i} \left( t \right)} \right| - \lambda_{i} } \right),} \hfill & \quad {\left| {c_{i} \left( t \right)} \right| \ge \lambda_{i} } \hfill \\ {0,} \hfill & \quad {\left| {c_{i} \left( t \right)} \right| < \lambda_{i} } \hfill \\ \end{array} } \right.\quad \left( {i = 1,2, \ldots ,k} \right),$$

where sgn(•) represents the symbolic function, *λ*_*i*_ denotes the threshold of the IMF component *c*_*i*_(*t*).

When 1 ≤ *i* ≤ 2, the noise energy of corresponding IMF component is larger, and the signal-to-noise ratio is lower. The threshold *λ*_*i*_ is taken as

11 $$\lambda_{i} = \hat{\sigma }\sqrt {2\ln \left( M \right)} ,$$

where $\hat{\sigma }$ represents the noise level estimation, $\hat{\sigma } = m/0.6745,$*m* is the median of absolute deviation for *c*_1_(*t*), *M* represents the sequence length.

When 2 ≤ *i* ≤ *k*, the useful signal energy of corresponding IMF component is close to the noise energy. The threshold should be reduced. So the threshold *λ*_*i*_ is taken as

12 $$\lambda_{i} = {{\hat{\sigma }\sqrt {2\ln \left( M \right)} } \mathord{\left/ {\vphantom {{\hat{\sigma }\sqrt {2\ln \left( M \right)} } {\ln \left( {i + 1} \right)}}} \right. \kern-0pt} {\ln \left( {i + 1} \right)}}.$$

#### Reconstruct the signal

Equation (4) is applied to the signal reconstruction. The reconstructed results *x*′(*t*) form a noise-removed observation series of dam safety.

## Actual case analysis

**Fig. 4:**
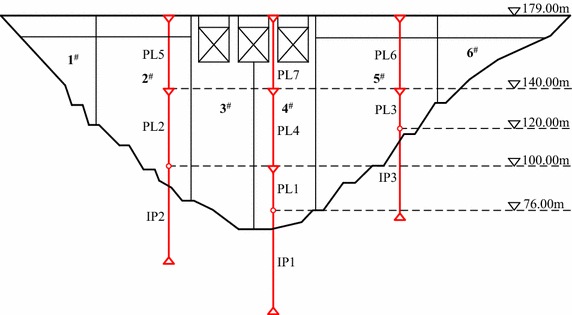
Layout of pendulum measurements observing horizontal displacement

**Fig. 5:**
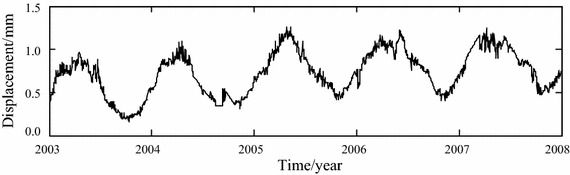
Original observations of horizontal displacement

One roller compacted concrete gravity dam called Mianhuatan in China is taken as an example. The maximum dam height is 113.0 m, the length of dam crest is 308.5 m, and the elevation of dam crest is 179.0 m. This dam consists of 6 dam sections which are numbered 1–6 from left bank to right bank. The normal storage water level and the check flood level are 173.00 and 177.80 m respectively. The dam construction officially began in April 1998, and the first unit was put into operation on April 29, 2001. The pendulum measurements in Fig. 4 were installed to observe the horizontal displacement of dam crest and dam body. The monitoring system was put into operation in October, 2002. Figure 5 shows the time curve of horizontal displacement along river direction, which is measured daily from January 1, 2003 to December 31, 2007 with the pendulum measurement PL6. It can be seen from Fig. 5 that obvious fluctuation with small amplitude appears in the observation series of horizontal displacement of No. 5 dam section crest. The proposed method is adopted to remove the noise of collected observations.

**Fig. 6:**
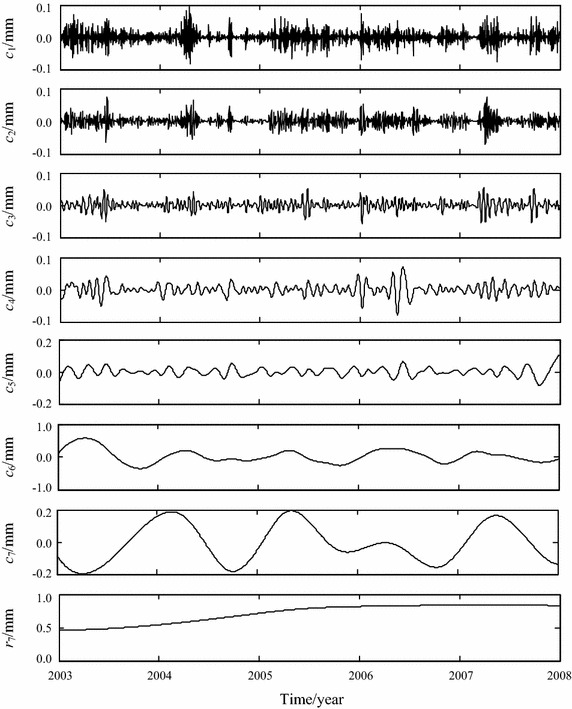
EEMD results of observation series

EEMD for the observation series shown in Fig. 5 is implemented. 7 IMF components, *c*_1_, *c*_2_,…, *c*_7_, and one residual *r*_7_ are obtained, as shown in Fig. 6.

**Fig. 7:**
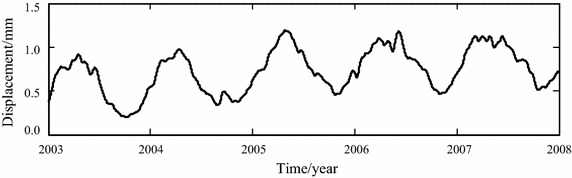
Noise-removed observation of horizontal displacement

The calculations with Eq. (9) implies that, when *k* = 3, *R*_*k*_ = 3.9 > *C* (*C* = 3). So the first 3 IMF components are selected to implement the noise removal operation respectively with the threshold 0.0468, 0.0468 and 0.0338. The sum of noise-removed components, other IMF components and the residual, namely noise-removed observation series, is shown in Fig. 7.

Comparison between Figs. 5 and 7 shows that after the EEMD-based noise removal is implemented, most of the fluctuations with small amplitude appearing in the original observation series have been filtered. The time-varying feature of horizontal displacement can be reflected more clearly.

To assess the noise removal performance of proposed method, the original and noise-removed observation series are taken to build the statistical models of horizontal displacement with the stepwise regression method. For the dam displacement caused by the action of water load, temperature load and other loads, such as large fluctuations in water level due to landslide induced tsunamis and submarine landslides impacting the dam (Pudasaini 2014; Kafle et al. 2016), it can be treated as the sum of hydrostatic pressure term, temperature term and time effect term. In the case study of this paper, the following factor set *F* is adopted to build the statistical model (Su et al. 2012, 2015).

13 $$F = \left[ {H,H^{2} ,H^{3} ,\sin \frac{2\pi t}{365},\cos \frac{2\pi t}{365},\sin \frac{4\pi t}{365},\cos \frac{4\pi t}{365},\ln \theta ,\theta } \right],$$

where *H* represents the upstream reservoir water depth, *t* denotes the cumulative days from the monitoring day to the beginning day, *θ* = *t*/100.

The statistical model can be described as follows (Su et al. 2012, 2015).

14 $$y^{{\prime }} = a_{0} + \sum\limits_{i = 1}^{3} {a_{i} H^{i} } {\kern 1pt} + \sum\limits_{i = 1}^{2} {\left[ {b_{1i} \sin \frac{2\pi it}{365} + b_{2i} \cos \frac{2\pi it}{365}} \right] + d_{1} \theta + d_{2} \ln \theta } ,$$

where *y*′ denote the model calculation, *a*_0_, *a*_*i*_, *b*_1*i*_, *b*_2*i*_, *d*_1_, *d*_2_ represent the regression coefficients.

**Fig. 8:**
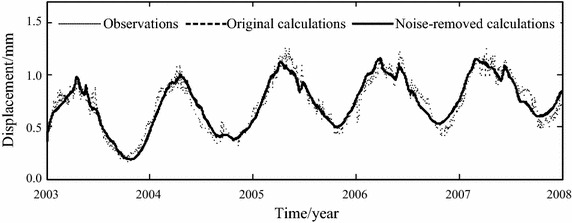
Calculated results of two statistical models

**Fig. 9:**
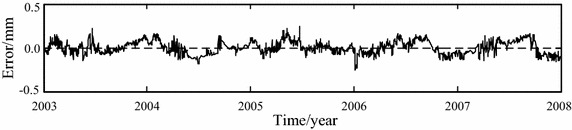
Modeling error of original observations-based statistical model

**Fig. 10:**
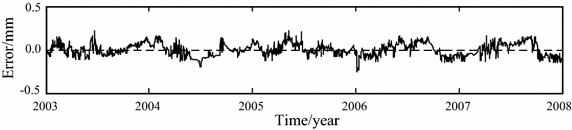
Modeling error of noise-removed observations-based statistical model

The original and noise-removed observation series from January 1, 2003 to December 31, 2006, which are shown in Figs. 5 and 7 respectively, are chosen to build the statistical models of horizontal displacement. The built models are used to forecast the horizontal displacement in 2007. The fitted and forecasted results of two models are shown in Figs. 8, 9 and 10. In this paper, the fitting and forecasting performances of built models are assessed using the squared correlation coefficient (*r*^2^) and the following mean square error (MSE).

15 $${\text{MSE}} = \frac{1}{l}\sum\limits_{i = 1}^{l} {\left( {y_{i}^{{\prime }} - y_{i} } \right)^{2} } ,$$

where *y*_*i*_ and *y*_*i*_′ denote the dam displacement observation and the model calculation respectively, *l* represents the number of measured values.

For the statistical model built based on the original observation series of horizontal displacement, its fitting MSE is 0.0051 and its forecasting MSE is 0.0073, its fitting *r*^2^ is 0.9536 and its forecasting *r*^2^ is 0.9250. For the statistical model built based on the noise-removed observation series of horizontal displacement, its fitting MSE is 0.0050 and its forecasting MSE is 0.0071, its fitting *r*^2^ is 0.9861 and its forecasting *r*^2^ is 0.9568. It can be seen that the noise removal improve the performance of built model.

## Conclusions

Considering the nonlinear and non-stationary characteristics of prototypical observations on dam safety, an EEMD-based method is introduced to remove noise from the original observation series with certain intermittency. Its basic principle and implement process are presented. To adapt the noise removal requirements of prototypical observations on dam safety, the key control parameters of EEMD algorithm are given and some improvement strategies are discussed.

The application example illustrates that the proposed method can filter the fluctuations with small amplitude appearing in the prototypical observation series on dam safety. The statistical model, which is built by choosing the noise-removed observations on dam safety, has better performance forecasting the dam behavior. Due to the high ability solving the mode mixing and endpoint effect problems, the EEMD-based method is more suitable for implementing the noise removal of prototypical observations on dam safety, particularly with certain intermittency.
